# Mitochondrial DNA quantification correlates with the developmental potential of human euploid blastocysts but not with that of mosaic blastocysts

**DOI:** 10.1186/s12884-023-05760-w

**Published:** 2023-06-15

**Authors:** Wen Luo, Yi-Min Zheng, Yan Hao, Ying Zhang, Ping Zhou, Zhaolian Wei, Yunxia Cao, Dawei Chen

**Affiliations:** 1 Department of Obstetrics and Gynecology, the First Affiliated Hospital of Anhui Medical University, No 218 Jixi Road, Hefei, 230022 Anhui China; 2 Anhui Provincial Engineering Technology Research Center for Biopreservation and Artificial Organs, Anhui Medical University, No 81 Meishan Road, Hefei, 230032 Anhui China; 3 NHC Key Laboratory of Study On Abnormal Gametes and Reproductive Tract (Anhui Medical University), No 81 Meishan Road, Hefei, 230032 Anhui China

**Keywords:** Mitochondrion, Mitochondrial genome, mtDNA quantification, Blastocyst, Embryo viability, Preimplantation genetic testing

## Abstract

**Purpose:**

We aimed to study the association between adjusted mtDNA levels in human trophectoderm biopsy samples and the developmental potential of euploid and mosaic blastocysts.

**Methods:**

We analyzed relative mtDNA levels in 2,814 blastocysts obtained from 576 couples undergoing preimplantation genetic testing for aneuploidy from June 2018 to June 2021. All patients underwent in vitro fertilization in a single clinic; the study was blinded—mtDNA content was unknown at the time of single embryo transfer. The fate of the euploid or mosaic embryos transferred was compared with mtDNA levels.

**Results:**

Euploid embryos had lower mtDNA than aneuploid and mosaic embryos. Embryos biopsied on Day 5 had higher mtDNA than those biopsied on Day 6. No difference was detected in mtDNA scores between embryos derived from oocytes of different maternal ages. Linear mixed model suggested that blastulation rate was associated with mtDNA score. Moreover, the specific next-generation sequencing platform used have a significant effect on the observed mtDNA content. Euploid embryos with higher mtDNA content presented significantly higher miscarriage rates and lower live birth rates, while no significant difference was observed in the mosaic cohort.

**Conclusion:**

Our results will aid in improving methods for analyzing the association between mtDNA level and blastocyst viability.

**Supplementary Information:**

The online version contains supplementary material available at 10.1186/s12884-023-05760-w.

## Introduction

Mitochondria are the core organelles of bioenergetics and affect most cellular functions. Each mitochondrion carries multiple copies of mitochondrial DNA (mtDNA), and variations in mtDNA copy number are observed in human tissues [1]. Alterations in mtDNA copy number in a cell impair mitochondrial function and contribute to the pathology of several diseases, such as chronic diseases, cardiovascular disease, lymphoma, breast cancer, and melanoma [2–4]. In human reproduction, changes in the mtDNA copy number are associated with maternal aging, decreased ovarian reserve, and inferior results following in vitro fertilization (IVF) [5–7].

Despite significant advancements in assisted reproductive technologies, little progress has been made on the clinical indicators used to determine embryo viability. Embryologists continue to rely on subjective and imprecise morphometric and morphological grading measures to select suitable embryos for transfer [8, 9]. Although preimplantation genetic testing for aneuploidy (PGT-A) enables the detection of euploid embryos and minimizes the risk of miscarriage while increasing live birth rates per transfer [10–12], a considerable proportion of transferred euploid embryos fail to result in a viable pregnancy, highlighting the fact that chromosomal normality is not the only factor that determines embryonic viability [13]. Another emerging problem regarding PGT-A is that a considerable proportion of samples with mosaicism readouts, which range from 20 to 25% [14], show the concurrence of both euploid and aneuploid cells within blastocysts [15]. Although mosaic embryos result in lower implantation and live birth rates than euploid embryos [13, 16], recent research has shown that mosaic embryos can mature into healthy euploid infants [17–21]. The characteristics that affect the clinical outcomes of mosaic embryos are of interest in human reproductive medicine.

As animal studies have revealed a strong link between mitochondria and reproductive function [22–24], interest in mitochondria in human reproduction has increased, along with the use of mitochondrial parameters to supplement currently available selection methods for predicting the viability of preimplantation embryos in IVF [6]. Several research groups have proposed using mtDNA as a prognostic indicator for embryo viability and embryo implantation potential [25–29]. Such findings are unexpected because, unlike cumulus cells, embryos with a greater implantation potential do not carry an increased mtDNA copy number [30, 31]. Conversely, blastocysts with a lower implantation potential—indicating that they are stressed and thus, less viable—show increased mtDNA replication or mitochondrial biogenesis. Although some research groups have not found such a correlation [32, 33], the true biological relevance of this concept and its potential utility in medically assisted reproduction as an additional indicator of embryo competency must be determined.

In this study, we investigated the values and distribution of mtDNA copy numbers in trophectoderm (TE) cells produced from human blastocysts under various ploidy circumstances (i.e., euploid, mosaic, and aneuploid) and the factors that might impact their final count to gain a better understanding of the factors impacting mtDNA values. We also explored whether a relationship exists among embryonal mtDNA copy number, embryo quality, and IVF outcomes in both euploid and mosaic blastocysts. This may aid in the assessment of mtDNA copy number values as a measure of embryo viability.

## Materials and methods

### Ethics approval

This study was approved by the Ethics Committee of the First Affiliated Hospital of Anhui Medical University, China (No. 20211217). This study was a retrospective analysis of archived data using clinical samples obtained from patients referred to the Reproductive Medical Center of Anhui Medical University for the purpose of PGT-A. Written informed consent for the analysis of archived data was obtained from patients with the approval of the Ethics Committee.

Embryos in this study were subjected to TE biopsy at the request of the patients for chromosomal aneuploidy assessment. For this purpose, the standard procedure in our laboratory involved whole-genome amplification (WGA; SurePlex, Illumina, CA, USA) followed by next-generation sequencing (NGS) on the Ion Proton (Thermo Fisher Scientific, MA, USA) or NextSeq550 (Illumina, CA, USA) platform. The experimental procedure described herein involved the analysis of archived data following the completion of PGT-A. The embryos were not subjected to additional manipulations, and the clinical treatment of the patients was not changed as a result of their participation in this study.

### Patients and samples

The study included 2,814 biopsied blastocysts from all 664 PGT-A cycles (576 couples, among whom five completed three cycles and 78 completed two cycles) performed from June 2018 to June 2021. Of the 2,814 biopsied samples subjected to NGS, 1,460 were processed on a semi-conduct sequencing platform (Ion Proton), whereas 1,354 were processed on an Illumina sequencing platform (NextSeq550). All samples were de-identified prior to the initiation of this study. Table 1 summarizes the characteristics of the study population, and no significant differences were observed between samples processed on different NGS platforms.

**Table 1:** Clinical characteristics of the patient population studied

**Characteristic**	**Mean** ± **SD**	
	**NextSeq550 Data**	**Proton Data**
Female age (years)	32.83 ± 5.41	31.67 ± 5.40
BMI (kg/m^2^)	22.34 ± 3.03	21.99 ± 2.84
Basal FSH (IU/L)	7.37 ± 2.19	7.28 ± 2.06
Basal LH (IU/L)	4.84 ± 2.93	5.54 ± 6.27
Gn administrated (IU)	2285.90 ± 716.99	2300.88 ± 727.31
Estradiol on trigger day (pmol/L)	12861.06 ± 5273.01	12909.12 ± 5057.72
Progesterone on trigger day (nmol/L)	4.29 ± 2.08	4.62 ± 2.31
Ovarian sensitivity index	7.54 ± 5.25	8.00 ± 5.90
Number of retrieved oocytes	15.12 ± 8.23	16.33 ± 8.96
Oocyte maturation rate (%)	76.21 ± 16.08	76.75 ± 16.83
Fertilization rate (%)	88.44 ± 14.73	89.74 ± 12.75
Rate of 2PN zygotes (%)	82.98 ± 17.80	82.32 ± 19.66
Cleavage rate (%)	98.46 ± 4.47	98.47 ± 5.11
Blastulation rate (%)	52.77 ± 24.49	51.55 ± 20.54
Male age (years)	34.56 ± 6.47	33.14 ± 5.90
Sperm concentration (mil/mL)	80.46 ± 81.62	84.47 ± 70.54
Rate of PR sperm (%)	41.31 ± 19.42	38.85 ± 19.84
Rate of anomaly sperm (%)	96.02 ± 2.31	95.96 ± 3.04
DFI of sperm (%)	19.19 ± 11.71	19.13 ± 12.72

*BMI* body mass index, *DFI* DNA fragmentation index, *FSH* follicle-stimulating hormone, *LH* luteinizing hormone, *Gn* gonadotropin, *PR* progressive motility, *SD* standard deviation

### Sample processing

Oocyte retrieval and intracytoplasmic sperm injection were performed as previously described [34]. For embryo micromanipulation and trophectoderm biopsy, embryos were transferred from blastomere medium (COOK, Sydney, Australia) to blastocyst medium (COOK, Sydney, Australia) at 10:00 on the third day after insemination and cultured until they reached the blastocyst stage on days 5 or 6. The biopsy was conducted between 11 a.m. and 12 p.m. on day 5 or 6. First, an enlarged blastocyst was attached with a holding pipette (Sunlight Medical Inc, Jacksonville, USA) at the 9 o'clock position, and then a laser was used to produce a tiny hole in the zona pellucida (Hamilton Thorne LYKOS, Beverly, MA, USA). Subsequently, the biopsy pipette was constantly pressed against the blastocyst's perimeter until the TE shrunk and detached from the inner surface of the zona pellucida. Multiple laser pulses were then utilized to bore a 5 μm hole at the far end of the zona pellucida from the inner cell mass (ICM). The biopsy pipette was then introduced into the blastocyst through the hole, and the constricted TE was aspirated under negative pressure. Some TE cells were simultaneously extracted from the hole, and a laser was used to chop TE cells along the flat mouth of the pipette. All procedures were carried out on a heated micromanipulator (ECLIPSE Ti2, Nikon, Japan). Following biopsy, the blastocysts were cryopreserved. The TE samples were placed in a 200μL PCR tube with 2 L phosphate-buffered saline (Thermo Fisher Scientific). According to the manufacturer's guidelines, the DNA isolated from TE samples was submitted to WGA using SurePlex (Illumina), followed by NGS, using the Ion Proton (Thermo Fisher Scientific) or NextSeq550 (Illumina) systems. The reads were aligned to the NCBI GRCh37 human reference genome using in-built analysis software and converted to BAM files.

### Chromosomal abnormality analysis

To analyze chromosomal abnormalities, the genome was partitioned into non-overlapping windows of constant size ranging from 600 kb to 1 Mb. Subsequently, a set of 30 reference values was constructed by averaging the proportion of mapped reads attributed to each window in a series of euploid samples. The ratio of the percentage of reads derived from a chromosomal window > 10 Mb in an embryo (test) sample to the reference value corresponding to the same window was calculated. Copy number variations with a length of > 4 Mb were considered positive. Ratios > 1.25 were associated with chromosomal duplication, whereas ratios < 0.75 were associated with deletion [35].

### Mosaicism determination

To determine mosaicism, read counts in each window (RC) were normalized to avoid the local GC content and RC biases induced by genomic mappability. Briefly, RCs were corrected using the observed coverage deviation for a specific GC percentage and mappability score using Eq. 1.

1 $$\overline{{\mathrm{RC} }_{i}}=\frac{\mathrm{m}}{{\mathrm{m}}_{\mathrm{GC}}}\cdot \frac{m}{{\mathrm{m}}_{\mathrm{MAP}}}$$

where, RCi denotes the read counts for the i-th window, mGC denotes the median read counts for all windows with the same GC percentage as the i-th window, mMAP denotes the median read counts for all windows with the same mappability score as the i-th window, and m denotes the overall median of all windows [36].

The hidden Markov model (HMM) was employed to determine mosaicism. The HMM matrix was established based on known negative samples, and eight chromosomal status codes were set as follows: 0, 5, 10, 15, 20, 25, 30, and 40, representing homozygous deletion, deletion mosaicism, haploid, deletion mosaicism, diploid, duplication mosaicism, triploid, and tetraploid, respectively. The transition probability between the same states of the initial matrix was 0.999, and the transition probability between different states was 0.025. The observed state transition probability matrix was calculated using the RC value for each window of the known negative sample. The state probability of each window was calculated using the above matrix and the corrected RC value of the sample to be tested. For windows that were reported as anomalies by HMM, the mosaic ratio was calculated using Eq. 2.

2 $$\mathrm{Ratio}=\frac{\left(\left|{\mathrm{RC}}_{\mathrm{j}}-{\mathrm{RC}}_{\mathrm{N}}\right|\right)}{10}\cdot 100 {\mathrm{RC}}_{\mathrm{N}}\in \left(\mathrm{10,20}\right)$$

where, RC_j_ is the read count of the j-th window and RC_N_ is the value of each window of the known negative sample.

Mosaicism of > 80% or < 20% cannot be distinguished from technical noise; therefore, samples with > 80% mosaicism were categorized as aneuploid, those with < 20% mosaicism were categorized as euploid, and those with mosaicism between 20 and 80% were categorized as mosaic. Mosaicism-positive chromosomes were analyzed in detail using our NGS validation data.

### Calibrated algorithm for mtDNA content

To calculate the mtDNA content, an optimized algorithm was used to process the output dataset generated by PGT-A analysis, which contained a mixture of mtDNA and nuclear DNA (nDNA) readings. To determine the relative copy number of mtDNA in embryos, the number of filtered reads mapped to the mitochondrial genome was divided by the number of reads mapped to the nuclear genome, and then multiplied by 100. This enabled batch normalization, thereby reducing the variability of NGS studies.

We extended the algorithm developed by Victor et al*.* [33]. Specifically, the total number of reads mapped to the mitochondrial genome (r_m_) was divided by the total number of reads mapped to the nuclear genome (r_n_). The corrected mtDNA score m_NGS_ was defined as the product of the r_m_ r_n_ dividant multiplied by the correction factor F_NGS_ (Eq. 3).

3 $${\mathrm{m}}_{\mathrm{NGS}}=\frac{{\mathrm{r}}_{\mathrm{m}}}{{\mathrm{r}}_{\mathrm{n}}}\cdot {\mathrm{F}}_{\mathrm{NGS}}$$

where, F_NGS_ considers two parameters to adjust for the number of cells examined: embryo sex (sex correction factor, SCF) and ploidy (ploidy correction factor, PCF) using Eq. 4.

4 $${\mathrm{F}}_{\mathrm{NGS}}=\mathrm{SCF}\cdot \mathrm{PCF}$$

where, SCF for female embryos equals 1 and SCF for male embryos equals 0.9842, because the length of male genomes is 98.42% that of female genomes. Aneuploid or mosaic embryos possess altered genetic material compared with euploid embryos, resulting in increased mtDNA copy numbers in monosomies and nullisomies, or decreased mtDNA levels in polysomies if not corrected. To correct for this, the mtDNA value for each embryo is multiplied by PCF based on the chromosomal composition and degree of mosaicism (Eq. 5). RGL represents the length of the reference genome, which is defined as the length of the diploid female at 6,072,607,692 bp. Parameter k represents the degree of mosaicism, which varies between 30 and 80%.

5 $$\mathrm{PCF}=\frac{\left(\mathrm{RGL}\pm \mathrm{Z}\right)\times \mathrm{k}+\left(\mathrm{RGL}\right)\times \left(1-\mathrm{k}\right) }{\mathrm{RGL}}$$

When embryos contain many lesions, the correction factors for each lesion should be multiplied. Thus, the corrected mtDNA score for embryos with aneuploidy mosaicism in more than one (n ≥ 1) chromosome segment was calculated using Eq. 6.

6 $${\mathrm{F}}_{\mathrm{NGS}}=\mathrm{SCF}\times \prod_{i=1}^{n}{\mathrm{PCF}}_{i}$$

### Statistical analysis

Statistical analysis was performed using SPSS 24 (IBM Corp., USA). Categorical data were compared using the Chi-squared test and Fisher’s exact test, where appropriate. Continuous data were compared using the Mann–Whitney *U* test and Wilcoxon signed-rank test, where appropriate. Linear regression analysis was applied to test the correlation between maternal age and mtDNA contents. Linear mixed models (LMMs) with a random cohort effect were used to test the effects of patient-specific characteristics, ovarian stimulation variables, and embryo parameters on the mtDNA content. Models were simplified by removing nonsignificant interaction terms based on a likelihood ratio test. Results were considered statistically significant at *P* < 0.05 and indicated high significance at *P* < 0.001.

## Results

### mtDNA content values differ between NGS platforms

**Fig. 1:**
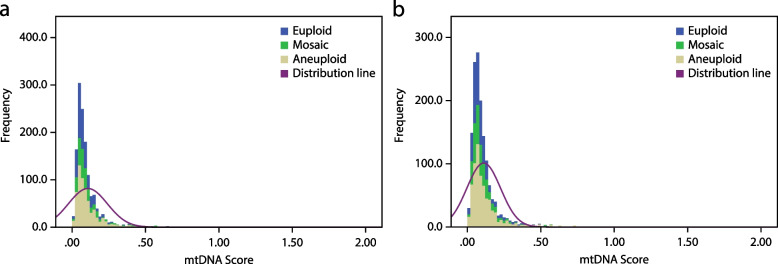
Distribution of mitochondrial DNA scores in trophectoderm of human blastocysts measured using next-generation sequencing on Illumina NextSeq550 platform (**a**) and semi-conduct Proton platform (**b**)

The mitochondrial DNA (mtDNA) scores obtained from both the NextSeq550 and Ion Proton platforms exhibited a non-normal distribution, as illustrated in Fig. 1a and b. Specifically, the data sourced from the NextSeq550 platform displayed a positive asymmetry of 6.242 ± 0.066 and kurtosis of 55.154 ± 0.133, while the data sourced from the Ion Proton platform exhibited a positive asymmetry of 5.450 ± 0.064 and kurtosis of 48.744 ± 0.128. The median and interquartile range (IQR) values for mtDNA scores obtained from NextSeq550 were 0.074 and 0.066, respectively. On the other hand, the median and IQR values for mtDNA scores obtained from Ion Proton were 0.082 and 0.072, respectively. Statistical analysis using the Mann–Whitney test revealed a significant difference between the mtDNA scores obtained from Ion Proton and NextSeq550 platforms (*P* < 0.001). To eliminate the possibility of differences arising from variations in samples, 100 randomly selected samples that were tested using the Ion Proton platform were re-examined using the NextSeq550 platform. The Wilcoxon Signed Ranks test still showed a significant difference between the mtDNA scores obtained from the two different sequencing platforms (*P* < 0.001).

### mtDNA contents are correlated with blastocyst ploidy status

**Fig. 2:**
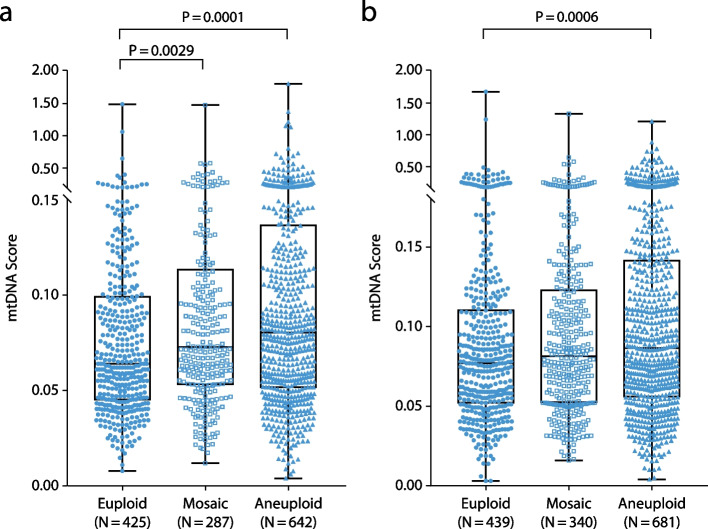
Mitochondrial DNA scores sorted by blastocyst ploidy result in statistically significant differences. **a** Next-generation sequencing data from Illumina NextSeq550 platform. **b** Next-generation sequencing data from semi-conduct Proton platform

**Fig. 3:**
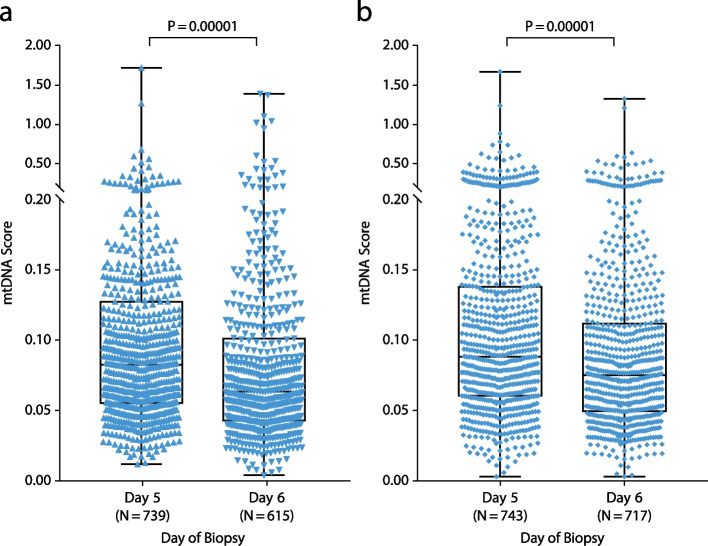
Mitochondrial DNA scores sorted by biopsy day result in statistically significant differences. **a** Next-generation sequencing data from Illumina NextSeq550 platform. **b** Next-generation sequencing data from semi-conduct Proton platform

For Nextseq550 data, the median value of euploid embryos was 0.064, compared with 0.080 for aneuploid embryos and 0.073 for mosaic embryos. For Ion Proton data, the median value of euploid embryos was 0.077, compared with 0.086 for aneuploid embryos and 0.081 for mosaic embryos. Differences between the euploid and aneuploid groups were significant for both NextSeq550 (*P* = 0.0001) and Ion Proton (*P* = 0.0006) data (Fig. 2a and b). NextSeq550 data revealed significant differences between mosaic and euploid samples (*P* = 0.0029), whereas no significant difference from mosaic samples was observed in either euploid or aneuploidy samples sourced from Ion Proton data (Fig. 2a and b). Day 5 embryos tended to carry higher levels of mtDNA than day 6 embryos (*P* = 0.00001 for both NextSeq550 and Ion Proton data) (Fig. 3a and b).

### Blastulation rate and day of biopsy, but not maternal age or embryo quality, are associated with mtDNA contents

**Fig. 4:**
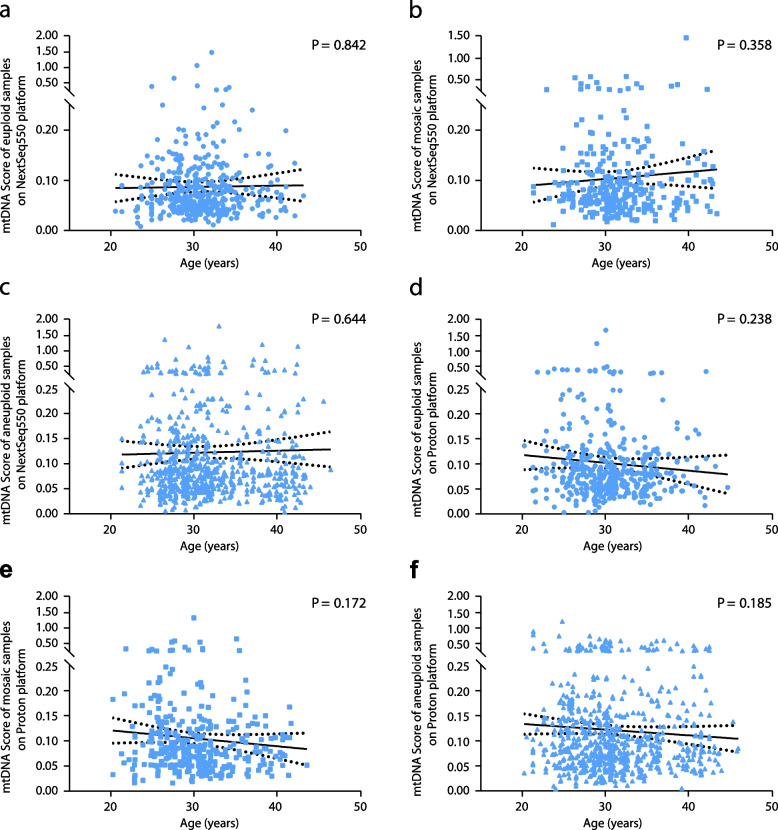
Mitochondrial DNA scores of blastocysts sorted by maternal age at the time of oocyte retrieval. Euploid (**a**), Mosaic (**b**) and Aneuploid (**c**) embryos measured using next-generation sequencing on Illumina NextSeq550 platform. Euploid (**d**), Mosaic (**e**) and Aneuploid (**f**) embryos measured using next-generation sequencing on semi-conduct Proton platform. Linear regression analysis results in statistically insignificant P values in all cohorts

Samples were divided into two groups based on maternal age above or below 35 years old at oocyte retrieval. Significant differences in the proportion of aneuploid embryos between both groups were observed using both NextSeq550 (*P* = 0.001) and Ion Proton (*P* = 0.005) data (Supplementary fig. S1). Linear regression analysis of mtDNA scores among euploid, mosaic, and aneuploid embryos revealed no difference between embryos derived from oocytes of different maternal ages for either NextSeq550 or Ion Proton data (Fig. 4a–f).

To analyze other factors that could be associated with embryonic mtDNA content, we employed an LMM to test the possible effects of patient-specific characteristics, ovarian stimulation variables, and embryo parameters on the mtDNA level of blastocyst TE biopsies. The model was simplified by removing nonsignificant interaction terms based on a likelihood ratio test. Blastulation rate (*P* = 0.021), day of biopsy (*P* = 0.000), ploidy of TE cells (*P* = 0.000), and NGS platform (*P* = 0.049) appeared to be associated with mtDNA score (Table 2). There was no evidence of an association between other patient-specific or embryo morphological variables and mtDNA scores.

**Table 2:** Linear mixed model to evaluate the effects of patient-specific variables on the mtDNA values of biopsied trophectoderm cells

	**Parameter**	**Estimate**	**SE**	**df**	**t-value**	***P*** ** value**
Fixed effect	Intercept	0.307413	0.073466	405.479	4.184	0.000
	Blastulation rate (%)	-0.106200	0.045261	85.164	-2.346	0.021
	Day of biopsy = Day 6	-0.388642	0.062201	1075.864	-6.248	0.000
	Day of biopsy = Day 5 ^a^	0	0			
	Nextseq550 platform	-0.075043	0.086246	203.331	-0.870	0.049
	Proton platform ^a^	0	0			
	Ploidy = euploidy	-0.287605	0.071493	1061.899	-4.023	0.000
	Ploidy = mosaicism	-0.106665	0.076040	1063.241	-1.403	0.161
	Ploidy = aneuploidy ^a^	0	0			
Random effect			Estimate	SE	Wald Z	*P value*
	Residual		0.846008	0.040767	20.752	0.000
	Intercept	Variance	0.161566	0.043503	3.714	0.000
	Blastulation rate (%)	Variance	0.053767	0.037718	1.426	0.045

^a^Reference group; Akaike Information Criterion = 3090.816

### mtDNA content is correlated with different clinical outcomes of euploid but not mosaic blastocysts

Among the 2,814 recruited samples, 868 embryos were transferred, 772 were euploid, and 96 were mosaic. To investigate the effect of mtDNA content on clinical outcomes, given the significant effect of biopsy day, ploidy, and NGS platform on the mtDNA copy number, we first evaluated the median and third quartile for different cohorts of embryos (Supplementary Table S1 and S2). Subsequently, we stratified embryos into groups by mtDNA content (above and below the median, or above and below the third quartile; Tables 3 and 4). Between the euploid and mosaic cohorts, there were no significant differences in positive beta-human chorionic gonadotropin (hCG), implantation, live birth, biochemical miscarriage, or miscarriage rates in embryos stratified by the median mtDNA content (Tables 3 and 4). The cutoff was set at the third quartile. Euploid embryos with higher mtDNA contents presented significantly higher miscarriage rates (*P* = 0.012) and lower live birth rates (*P* = 0.024), whereas no significant difference was observed in the mosaic cohort (Tables 3 and 4).

**Table 3:** Analysis of clinical outcomes of euploid 6 embryo transfers

		**Embryos transferred, n**	**Positive beta-hCG, n (%)**	***P *** **value**	**Biochemical miscarriage, n (%)**	***P*** ** value**	**Implantation, n (%)**	***P *** **value**	**Miscarriage n (%)**	***P *** **value**	**Live Birth, n (%)**	***P *** **value**
mtDNA score	< median	368	246 (66.8)		21 (8.5)		225 (61.1)					
	≧median	404	261 (64.6)	0.512 ^a^	22 (8.4)	0.965 ^a^	239 (59.2)	0.574^a^	42 (17.6)	0.395 ^a^	19^1^ (48.8)	0.344 ^a^
	< 3^rd^ quartile	572	382 (66.8)		32 (8.4)		350 (61.2)				302 (52.8)	
	≧3rd quartile	200	125 (62.5)	0.272 ^a^	11 (8.8)	0.883 ^a^	114 (57.0)	0.298 ^a^	27 (23.7)	0.012^a*^	87 (43.5)	0.024^a*^
ICM grade	A	140	101 (72.1)		5 (5.0)		96 (68.6)		11 (11.5)		85 (60.7)	
	B	625	401 (64.2)		37 (9.2)		364 (58.2)		64 (17.6)		300 (48.0)	
	C	7	5 (71.4)	0.189 ^a^	1 (20.0)	0.251 ^a^	4 (57.1)	0.077 ^a^	0 (0.0)	0.248 ^a^	4 (57.1)	0.023^a*^
Trophectoderm grading	A	100	69 (69.0)		10 (14.5)		59 (59.0)		10 (16.9)		49 (49.0)	
	B	596	401 (67.3)		31 (7.7)		370 (62.1)		57 (15.4)		313(52.5)	
	C	76	37 (48.7)	0.004^a**^	2 (5.4)	0.138 ^a^	35 (46.1)	0.026^a*^	8 (22.9)	0.505 ^a^	27 (35.5)	0.020^a*^
Day of embryo development	D5	533	373 (70.0)		29 (7.8)		344 (64.5)		56 (16.3)		288 (54.0)	
	D6	239	134 (56.1)	< 0.001^a***^	14 (10.4)	0.344 ^a^	120 (50.2)	< 0.001^a***^	19 (15.8)	0.909 ^a^	101 (42.3)	0.003^a**^
Age of oocytes	< 35	635	423 (66.6)		36 (8.5)		387 (60.9)		61 (15.8)		326 (51.3)	
	≧35	137	84 (61.3)	0.236^a^	7 (8.3)	0.958 ^a^	77 (56.2)	0.304 ^a^	14 (18.2)	0.598 ^a^	63 (46.0)	0.256 ^a^
Endometris thickness	< 10 mm	384	242 (63.0)		24 (9.9)		218 (56.8)		41 (18.8)		177 (46.1)	
	≧10 mm	388	265 (68.3)	0.123 ^a^	19 (7.2)	0.267 ^a^	246 (63.4)	0.060 ^a^	34 (13.8)	0.145 ^a^	212 (54.6)	0.018^a*^

^1^ *ICM* Inner cell mass. The grading system employed here is Gardner blastocyst grading system. *P < 0.05; **P < 0.01; ***P < 0.001^a^Pearson Chi-Square test

**Table 4:**
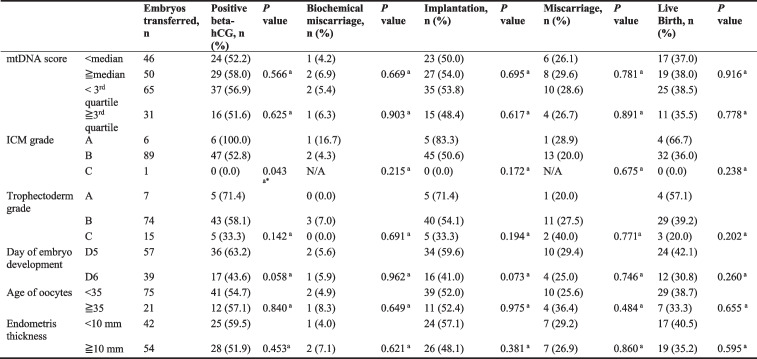
Analysis of clinical outcomes 8 of mosaic embryos

*ICM* Inner cell mass. The grading system employed here is Gardner blastocyst grading system

^*^P < 0.05

^a^ Pearson Chi-Square test

Furthermore, we investigated the factors associated with the viability of euploid and mosaic embryos. In the euploid cohort, a better ICM grade was associated with higher live birth rate (*P* = 0.023) (Table 3); a better trophectoderm grade was associated with a higher positive beta-hCG rate (*P* = 0.004), implantation rate (*P* = 0.026), and live birth rate (*P* = 0.020) (Table 3). Compared to blastocysts biopsied on day 5, blastocysts biopsied on day 6 were associated with a lower positive beta-hCG rate (*P* < 0.001), implantation rate (*P* < 0.001), and live birth rate (*P* = 0.003) (Table 3). Endometrial thickness was associated with higher live birth rate (*P* = 0.018) (Table 3). Oocyte age were not associated with the outcomes of euploid embryos (Table 3). In the mosaic cohort, we observed no significant associations between the aforementioned factors and outcomes (Table 4).

### Parameters of mosaicism affecting clinical outcomes

Mosaic embryos were associated with poorer outcomes than euploid embryos: positive beta-hCG rates, 55.2% versus 65.7% (*P* = 0.043); implantation rate, 52.1% versus 60.1% (*P* = 0.132); miscarriage rate, 28.0% versus 16.2% (*P* = 0.036); live birth rate, 37.5% versus 50.4% (*P* = 0.017; Table 5). We further investigated the potential effects of mosaic parameters on clinical outcomes. In our dataset, blastocysts with only whole chromosome mosaicism were associated with a considerably higher miscarriage rate (*P* = 0.005) and lower rate of live birth (*P* = 0.064) than euploid blastocysts, whereas exclusively sub-chromosomal mosaicism showed no significant difference compared with euploid blastocysts (Table 5). Notably, blastocysts carrying a single mosaic lesion site were associated with a higher miscarriage rate (*P* = 0.021) and lower livebirth rate (*P* = 0.046) than euploid blastocysts, whereas no significant results were observed in blastocysts carrying multiple mosaic lesion sites compared with euploid blastocysts (Table 5). The degree of mosaicism, which is an estimate of the proportion of aneuploid cells in the biopsy sample, appeared to be associated with different rates of positive beta-hCG (*P* = 0.005), implantation (*P* = 0.007), miscarriage (*P* = 0.027) and live birth (*P* = 0.002) (Table 5).

**Table 5:**
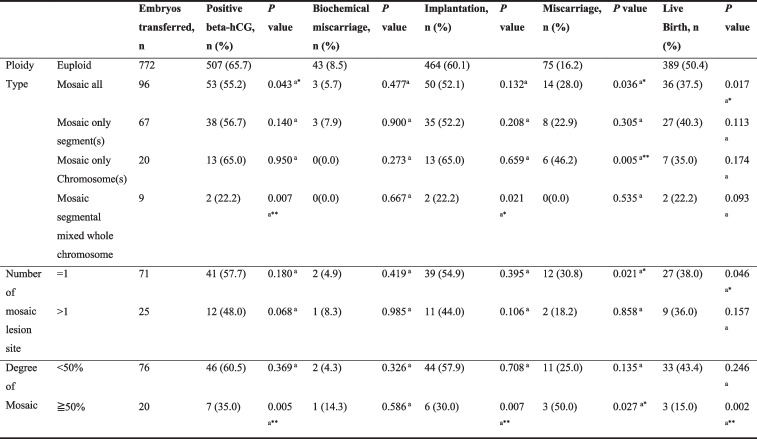
Analysis of mosaic parameters affecting clinical outcomes of mosaic 10 embryos transfer

^*^ *P* < 0.05. **P < 0.01

^a^Compared with euploid embryos

## Discussion

In the mid-1990s, dysfunction of mitochondrial bioenergetics was shown to result in poor oocyte quality, failure of fertilization and embryonic development, chromosomal aberrations, and poorer clinical outcomes after IVF [5, 7, 26–29, 37, 38]. Since then, mtDNA copy number has garnered considerable attention and been recommended as a predictor of embryo viability. However, inconsistencies exist across studies, resulting in contradictory outcomes [1, 5, 6, 25, 26, 28, 29, 32, 39–47].

Concerns have been raised regarding the reliability of the technology used to determine the mtDNA content of embryos across different centers, as well as the subsequent effect on treatment outcome [1]. In the present study, mtDNA content was determined using low-coverage NGS in conjunction with an internally validated method. Victor et al*.* [33] proposed autosome corrections, normalization of mtDNA against a multicopy nDNA sequence, and changes in chromosomal copy number and sex chromosomes to indicate a more accurate composition of mtDNA. We extended the algorithm developed by Victor et al*.* [33] to consider the heterogeneity of embryonic genomes to accurately evaluate mtDNA content. In concordance with the results of de Los Santos et al*.*and Lee et al*.* [41, 44], the adjusted mtDNA copy values followed a positive asymmetry distribution with high kurtosis. According to this distribution model, a large amount of data falls within a narrow range of extremely low values, whereas a small amount of data falls within an extremely high value range (e.g., five or more standard deviations from the mean). Given that data from both the NextSeq550 and Ion Proton platforms presented similar positive asymmetry distributions with high kurtosis, this is unlikely to be explained by a technical issue or bias error. It is possible that most embryos must meet particular mtDNA content requirements to develop into blastocysts. This also refers to a possible bottleneck during fertilization and embryo development, as has been previously suggested, because only oocytes carrying specific mitochondrial baggage are acceptable for fertilization and further embryo development [48–50].

To our knowledge, this study is the first to compare mtDNA levels between different NGS platforms and to demonstrate variations in mtDNA measurements across different sequencing technologies. Low-coverage whole genome sequencing relies on analyzing the depth of coverage of sequencing reads at different regions of the genome, and the relative mtDNA copy number is directly related to the number of filtered reads mapped to the mitochondrial genome. Given the nature of low coverage sequencing (typically 0.1-1x) and the length of the mitochondrial genome (16,535 base pairs), it is reasonable to expect different relative mtDNA levels across sequencing platforms with varying read lengths. The average read length of the Ion Proton platform is 200–400 base pairs, while that of the NextSeq550 platform is 2 × 75 base pairs.

In both datasets, the aneuploid embryos presented significantly larger quantities of mtDNA than the euploid embryos. This has been a topic of debate in previous reports. Interestingly, we used the same technology (NGS) and applied the same mathematical adjustment to mtDNA levels as Victor et al*.* [33] but obtained different results. A possible explanation is that the statistical analysis was conducted inappropriately in the study by Victor et al*.* [33]. As previously shown, the distribution of mtDNA is not normal; therefore, a non-parametric analysis should be used rather than a parametric analysis [33]. Additionally, various confounding variables, including facility-specific variance in IVF methodology, patient-specific features, technical bias, and sample preparation and storage, could lead to discrepant results [41, 44, 51]. Thus, we employed the LMM to analyze LMM statistics for the effect of confounding factors on mtDNA quantification in patient-specific layers.

We examined the effect of oocyte age on mtDNA content in euploid, mosaic, and aneuploid cohorts and observed no impact of age in either cohort. LMM, which considered the cluster effect of the embryos belonging to particular patients, validated this insignificance. This issue is challenging because several groups have reported contradictory results [26, 28, 44] or no effect [33, 41]. The lack of concordance among groups could be attributed to the various methods used to examine the data in the study. Based on our approach, linear regression analysis was performed separately in cohorts with different ploidy statuses. Furthermore, results from both the non-parametric test and LMM demonstrated that aneuploid embryos presented larger mtDNA quantities than euploid embryos. As the proportion of aneuploidy is positively correlated with oocyte age, it is not surprising that some previous studies [26, 28, 44] obtained positive results when analyzing the aging effect on mtDNA content without stratifying the study population by ploidy status.

Supporting the findings of previous studies [39, 43, 45], mtDNA content was significantly lower in samples biopsied on day 6 than in those biopsied on day 5. Replication of embryonic mtDNA in the blastocyst TE occurs after the tissue has differentiated and is committed to placental development [52–55]. Replication of mtDNA in the ICM occurs later in development, perhaps correlating with the loss of pluripotency [46] (see also Fragouli et al. 2017b) [25, 54]. Accordingly, the level of mtDNA significantly decreased during preimplantation development owing to the dilution effect caused by cell division. Consequently, by day 6 of development, each embryonic cell should possess only a few copies of mtDNA.

The results of the LMM suggested that patients with higher blastulation rates were likely to carry low levels of mtDNA. As embryos do not express the replication factors required to synthesize mitochondria until after implantation, without new mitochondrial biosynthesis, the total number of mitochondria within each blastomere declines owing to dilution [54, 56, 57].

Ho et al. [58] reported that mtDNA content in day 6 blastocysts increases as the number of hours needed to reach the blastocyst stage increases. It is reasonable to extrapolate that in patients with developmentally delayed or arrested embryos, cells may divide more slowly, resulting in a higher mtDNA content in trophectoderm cells. As the main cause of developmental delay or arrest is the activation of the mitotic checkpoint triggered by aneuploidy [59, 60], and euploid embryos have a higher blastulation rate [61], aneuploid TE cells bear a considerably higher mtDNA content than euploid TE cells. Although higher mtDNA contents are associated with aneuploid blastocysts, factors determining differences in mtDNA quantity may exist in oocytes prior to fertilization. Over half of the aneuploidies detected in blastocysts are the product of aberrations during female meiosis [62–64], suggesting that variables predisposing to meiotic aneuploidy in oocytes also affect mtDNA levels later in embryonic development.

Whether there is an association between mtDNA copy number and embryo viability remains a matter of debate. Previous studies have produced conflicting results on whether mtDNA copy number could [1, 5, 6, 25–29, 32, 33, 39–46] be a useful biomarker for embryo viability. One scenario that reconciles conflicting reports is an inappropriate method of analysis. As shown in the present study, biopsy day, ploidy, and NGS platform have a significant effect on mtDNA content value; therefore, it is inappropriate to establish a threshold of mtDNA level by treating the dataset as uniform, as done in previous studies [25, 26, 33].

By setting the cutoff point according to the mtDNA content distribution in the population stratified by these aforementioned factors, our observations suggest that euploid embryos with mtDNA contents above the third quartile threshold have a significantly higher chance of miscarriage and, consequently, a lower live birth rate. Our data provide strong support for the potential use of mtDNA quantity as a modality to predict euploid embryos with low viability. The reason for the increased mtDNA levels in certain non-viable blastocysts is unknown. One hypothesis is that increasing mtDNA levels are associated with increased energy requirements associated with increased metabolic activity, which may reflect the response of an embryo to a stressor. Alternatively, certain embryos may increase their mtDNA levels to compensate for mitochondrial deficiency. Mutations in mitochondrial or nuclear genomes can result in impaired organelle function [65, 66]. Further research into the molecular mechanisms that cause a rise in mtDNA content in some embryos will be essential to elucidate this phenomenon.

PGT remains one of the most intensely contested procedures in reproductive medicine. Previous study has suggested a possible association of embryo biopsy with an increased risk of hypertensive disorders of pregnancy and adverse perinatal outcomes [67]. The consequences of the embryo biopsy itself on obstetric outcomes remain uncertain and cannot be excluded, which could eventually represent a source of bias (overestimation of adverse effects in euploid which usually do not undergo biopsy). The new challenge in PGT development is the non-invasive approach (niPGT) involving analysis of cell-free DNA obtained from the spent culture media to limit all possible impairments associated with embryo biopsy. It is plausible that such a non-invasive approach, with a lower risk of damage to the embryos, could limit potential obstetric, neonatal, and long-term complications in children born after embryo biopsy for PGT [67].

Recently, mosaic blastocysts are extensively investigated and transferred, showing mounting evidence of their capacity to result in healthy live births. Proposed algorithms have emerged to prioritize their transfer based on specific aneuploidy types [68, 69]. To the best of our knowledge, this is the first study to evaluate the association between mtDNA content and embryo viability in a mosaic cohort. Although mosaic blastocysts exhibiting mtDNA copy numbers above the established threshold (the third quartile) displayed a propensity towards lower implantation and live birth rates, no statistically significant differences in clinical outcomes were found between mosaic blastocysts with different mtDNA levels. Notably, mosaic parameters, including the type of mosaicism, number of mosaic lesion sites, and degree of mosaicism, demonstrated associations with different clinical outcomes for mosaic embryos. This observation suggests that the impact of mitochondrial DNA (mtDNA) on the viability of mosaic embryos may be relatively modest in comparison to the prevailing influence of genetic abnormalities. Alternatively, it is plausible that the genetic abnormalities themselves could be linked to inherent alterations in mtDNA. Due to the limited sample size in our study, it poses a challenge to establish a robust statistical framework and yield definitive outcomes when analyzing the influence of mtDNA quantifications on the developmental potential of mosaic embryos while stratifying the cohort based on mosaic parameters (i.e., type of mosaicism, number of mosaic lesion sites, and degree of mosaicism). Therefore, future investigations utilizing longitudinal data and larger sample sizes encompassing neonatal outcomes will be pivotal in advancing our comprehension of the reproductive potential associated with mosaic embryos.

Furthermore, obstetric outcomes of pregnancies resulting from mosaic blastocyst transfers have gained increasing attention, raising concerns about the incidence and impact of confined placental mosaicism (CPM) in these pregnancies. CPM has recently been identified as a possible cause of small-for-gestational-age (SGA) infants [70]. Similar to the developmental potential, specific types of aneuploidy in CPM, such as trisomy of chromosome 16, appear to be associated with a higher incidence of adverse pregnancy outcomes [71]. Importantly, studies have highlighted the presence of altered mitochondrial expression and function at the placental and neonatal levels in cases of intrauterine growth restriction (IUGR) [72, 73], which can arise from hypoxic conditions. This suggests that evaluating the content of mitochondrial DNA (mtDNA) in mosaic blastocysts may not only serve as an indicator of embryo viability but also shed light on the hidden molecular pathways and mechanisms that impact placental function and its role in fetal growth.

Our study’s potential limitations include the fact that it is an observational study with a relatively small number of embryo transfers and a lack of generalizability due to the data being collected from a single location. As it is reported, the proportion of embryos with higher mtDNA levels varies between clinics, and the variation appears to be due to clinic-specific factors rather than differences in patient demographics [25]. Therefore, it is difficult to establish a universal numeric threshold for mtDNA levels as a predictor of poor clinical outcomes. Our findings suggest that a therapeutic application could be the inclusion of mtDNA copy number analysis into standard NGS genetic assays. By establishing a statistical baseline for mtDNA levels at a single center, the third quartiles of certain data may have predictive significance in in distinguishing between embryos capable of sustaining pregnancy and those that are not. Nonetheless, given that mtDNA content measurement for embryos that already have undergone PGT-A does not require any additional laboratory operation but only bioinformatic analysis, cost-effectiveness remains positive.

## Conclusion

In conclusion, corrected mtDNA content was significantly lower in euploid blastocysts than in aneuploid blastocysts in human embryos. Moreover, biopsy day, ploidy, and NGS platform were also found to exert a significant effect on mtDNA content, resulting in improved methods for analyzing the association between mtDNA level and blastocyst viability. These findings add to the growing body of data supporting the potential utility of mtDNA measurement as a biomarker of euploid embryo viability. The clinical relevance of our findings is the integration of mtDNA copy number analysis into standard genetic investigations conducted by NGS. Finally, further studies on mitochondrial function and randomized clinical trials are required to better understand the role of mtDNA in embryo development and clinical utility.

## Supplementary Information

**Additional file 1: ** **Supplementary Fig. S1.** Samples were divided into two groups based on maternal age above or below 35 years old at oocyte retrieval. Significant differences in the proportion of aneuploid embryos between both groups were observed using both NextSeq550 (P = 0.001) and Ion Proton (P = 0.005). **Supplementary Table S1.** Percentiles of mtDNA scores measured on the Illumina NextSeq 550 platform. **Supplementary Table S2.** Percentiles of mtDNA scores measured on the Ion Proton platform.

## Data Availability

The datasets used and/or analyzed during the current study are available from the corresponding authors upon reasonable request.
