# Supplementary material for: Mitochondrial DNA quantification correlates with the developmental potential of human euploid blastocysts but not with that of mosaic blastocysts
Source: BMC Pregnancy Childbirth. 2023 Jun 15;23:447. doi: 10.1186/s12884-023-05760-w (PMC10273751; doi:10.1186/s12884-023-05760-w)
Supplement: Supplementary file 1 — Additional file 1: Supplementary Fig. S1. Samples were divided into two groups based on maternal age above or below 35 years old at oocyte retrieval. Significant differences in the proportion of aneuploid embryos between both groups were observed using both NextSeq550 (P = 0.001) and Ion Proton (P = 0.005). Supplementary Table S1. Percentiles of mtDNA scores measured on the Illumina NextSeq 550 platform. Supplementary Table S2. Percentiles of mtDNA scores measured on the Ion Proton platform. [file 12884_2023_5760_MOESM1_ESM.pdf]

Supplementary Information

Mitochondrial DNA Quantification Correlates with the Developmental Potential of Human Euploid Blastocysts but not with that of Mosaic Blastocysts

W. Luo[a,c], YM.Zheng [a,b],Y. Hao[a,c], Y. Zhang[a], P. Zhou[a,b,c], Z. Wei[a,b,c], Y. Cao[a,b,c]* , D. Chen[a,b]*

[a]Department of Obstetrics and Gynecology, the First Affiliated Hospital of Anhui Medical University, no 218 Jixi Road, Hefei 230022, Anhui, China

[b]NHC Key Laboratory of Study on Abnormal Gametes and Reproductive Tract (Anhui Medical University), no 81 Meishan Road, Hefei 230032, Anhui, China

[c]Anhui Provincial Engineering Technology Research Center for Biopreservation and Artificial Organs, Anhui Medical University, no 81 Meishan Road, Hefei 230032, Anhui, China

***Corresponding authors**

chendawei0930@163.com (D.C.) (ORCID ID: 0000-0002-8201-0549)

caoyunxia6@126.com (Y.C.) (ORCID ID: 0000-0002-8715-0874)

Supplementary Fig. S1

Samples were divided into two groups based on maternal age above or below 35 years old at oocyte retrieval. Significant differences in the proportion of aneuploid embryos between both groups were observed using both NextSeq550 (P = 0.001) and Ion Proton (P = 0.005) .

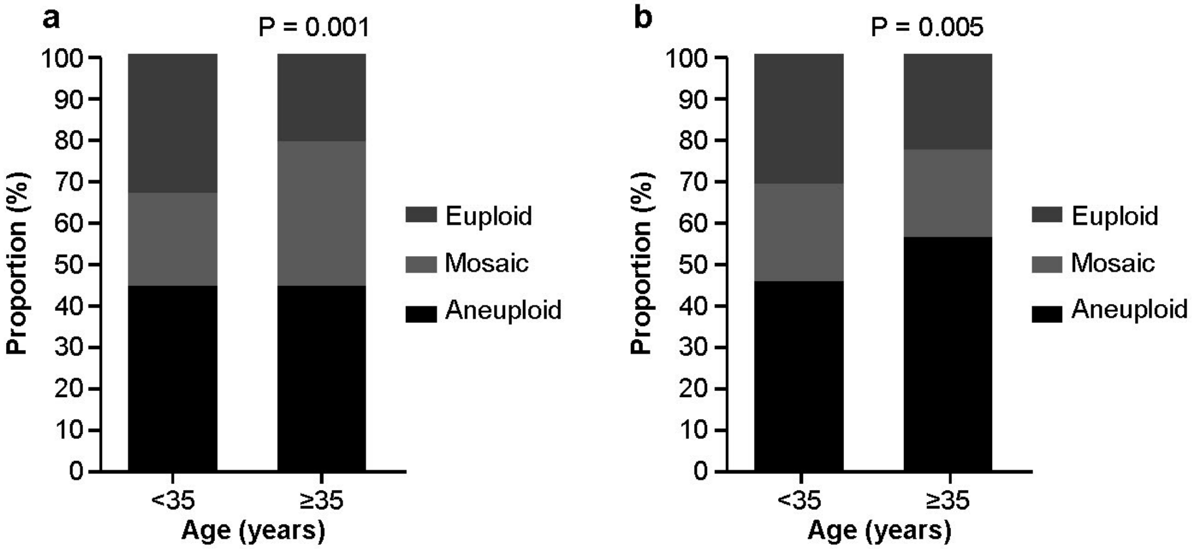

Supplementary Table S1. Percentiles of mtDNA scores measured on the Illumina NextSeq 550 platform

Ploidy	Biopsy day	Median	Third quartile	IQR
Euploid	Day 5	0.071847	0.107000	0.054918
	Day 6	0.054066	0.081197	0.041197
Mosaic	Day 5	0.082828	0.130708	0.075445
	Day 6	0.070299	0.101345	0.055824

Note: IQR, Interquartile range

Supplementary Table S2. Percentiles of mtDNA scores measured on the Ion Proton platform

Ploidy	Biopsy day	Median	Third quartile	IQR
Euploid	Day 5	0.083000	0.121058	0.063889
	Day 6	0.070000	0.098804	0.053349
Mosaic	Day 5	0.085973	0.128359	0.069694
	Day 6	0.077440	0.115611	0.064427

Note: IQR, Interquartile range
